# The Inhibition of the Inducible Nitric Oxide Synthase Enhances the DPSC Mineralization under LPS-Induced Inflammation

**DOI:** 10.3390/ijms232314560

**Published:** 2022-11-23

**Authors:** Amelia Cataldi, Rosa Amoroso, Viviana di Giacomo, Susi Zara, Cristina Maccallini, Marialucia Gallorini

**Affiliations:** Department of Pharmacy, University “G. d’Annunzio” of Chieti-Pescara, 66100 Chieti, Italy

**Keywords:** dental pulp stem cells, differentiation, inducible Nitric Oxide Synthase, inflammation, LPS, mineralization, nitric oxide, inhibitors, CD73, IL-6, VEGF

## Abstract

Nitric oxide (NO) is a key messenger in physiological and pathological processes in mammals. An excessive NO production is associated with pathological conditions underlying the inflammation response as a trigger. Among others, dental pulp inflammation results from the invasion of dentin by pathogenic bacteria. Vital functions of pulp mesenchymal stem cells (DPSCs, dental pulp stem cells), such as mineralization, might be affected by the inducible NOS (iNOS) upregulation. In this context, the iNOS selective inhibition can be considered an innovative therapeutic strategy to counteract inflammation and to promote the regeneration of the dentin-pulp complex. The present work aims at evaluating two acetamidines structurally related to the selective iNOS inhibitor **1400W**, namely **CM544** and **FAB1020**, in a model of LPS-stimulated primary DPSCs. Our data reveal that **CM544** and even more **FAB1020** are promising anti-inflammatory compounds, decreasing IL-6 secretion by enhancing CD73 expression-levels, a protein involved in innate immunity processes and thus confirming an immunomodulatory role of DPSCs. In parallel, cell mineralization potential is retained in the presence of compounds as well as VEGF secretion, and thus their angiogenetic potential. Data presented lay the ground for further investigation on the anti-inflammatory potential of acetamidines selectively targeting iNOS in a clinical context.

## 1. Introduction

The free radical nitric oxide (NO) is a ubiquitous molecular messenger which plays a key role in various physiological as well as pathological processes in mammals, bacteria, and plants. Low NO levels are generally associated with its homeostatic actions, which include immune function, control of the vascular tone and platelet aggregation, modulation of neurotransmission and memory [1]. Conversely, an excessive production of NO has been shown to be involved in immunological disorders, pain, neurological diseases, atherosclerosis, and cancer [2]. NO is endogenously synthesized by Nitric Oxide Synthases (NOSs), a family of three isoenzymes, the constitutively expressed neuronal NOS (nNOS), endothelial NOS (eNOS) and the inducible iNOS. In contrast to the constitutive NOS isoforms, the inducible produces high NO levels for sustained time periods and its activity is calcium independent. NO produced by iNOS proved to be crucial in nonspecific host defense and innate immunity, being cytotoxic against pathogen as well as tumor cells [3]. However, iNOS can promote a prolonged release of reactive nitrogen species (RNS), responsible for the damage of a wide variety of biomolecules, causing NO-related inflammatory and autoimmune diseases [4].

Dental pulp inflammation (pulpitis) results from the activation and interaction of host immune processes in response to physiochemical stimuli or both Gram-positive and Gram-negative bacteria and their toxins. The invasion of dentin by pathogenic bacteria, releasing their products across dentinal tubules, triggers the activation of innate immune responses in the dental pulp complex. These responses are mediated by Toll-like receptors (TLR) on the surface of cells of the dental pulp such as odontoblasts, pulp fibroblasts, dendritic cells and macrophages [5]. The TLR-binding starts the NF-κB (nuclear factor-κB)-dependent signaling cascade which is regulated by reactive oxygen (ROS) or RNS, such as hydrogen peroxide or NO produced by the iNOS [6,7]. The production of NO or RNS species serves to induce killing or stasis of the invading microorganisms, but an excessive stimulation of NO synthesis triggered by iNOS may also lead to cytotoxicity towards the host tissues, mediating the pathological effects of cytokines and pro-inflammatory enzymes [8]. Resident mesenchymal stem cells inside the dental pulp, i.e., dental pulp stem cells (DPSCs), are vulnerable to bacterial product-triggered infections and respond by secreting inflammatory mediators and with dysregulated mineralization [9]. It has been recently reported that both these processes might be prone to a disturbance of the redox homeostasis, and oxidative and nitrosative stress can influence vital functions of human dental pulp cells and their mineralization potential [10].

Based on its critical role in DPSCs, the modulation of iNOS has been proposed as a valuable strategy to prevent the irreversible damage to the dental pulp tissue and to promote the regeneration of dentin-pulp complex [11,12]. However, only few studies have been published to date, which mainly investigated the effects of unselective, or toxic, iNOS inhibitors. Moreover, inside the dental pulp tissue, endogenous NO is generated mainly by immunocompetent cells and dental pulp cells, and mediates not only inflammatory/immune activities, but also signaling cascades that regulate tissue repair and reconstruction, indicating its involvement in both tissue destruction and regeneration [11]. Basically, controlled, low levels of NO derived from the constitutive NOSs, mainly the eNOS, are essential under dental pulp physiological conditions, given the role played by NO in angiogenesis, tissue regeneration and perfusion [7,13]. As a consequence, it is essential not to inhibit the constitutive NOS when targeting the iNOS.

**Figure 1:**
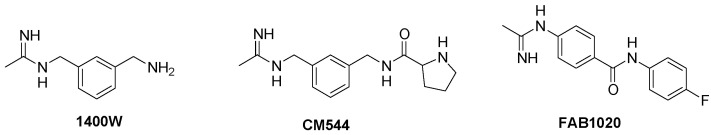
Chemical structure of iNOS inhibitors.

Different NOS inhibitors have been published in the last thirty years [14], but none of them have passed clinical trials. Therefore, there is still a need for safe molecules with appropriate tailored selectivity toward the inducible isoform. In this research context we have previously disclosed the acetamidines **CM544** and **FAB1020**, which are two compounds structurally related to the iNOS inhibitor **1400W** (Figure 1) [15,16]. These two acetamidines inhibit the iNOS with high potency and selectivity of action with respect to the constitutive NOSs, and, remarkably, they showed an ameliorated biological profile as anticancer, neuroprotective and immunomodulatory agents, in comparison with **1400W** [17,18,19]. The lipophilicity of **CM544** and **FAB1020** also appears somewhat increased with respect to the very polar and poorly cell-permeable **1400W** (Table 1).

Considering the close link between inflammation and the induction of regenerative processes in the dental pulp, as well as the lack of pre-clinical data on the potential therapeutic role that selective iNOS inhibitors might play in in vitro models of pulpitis, this work aims at studying the effects played by the two promising compounds **CM544** and **FAB1020** in LPS-stimulated DPSCs compared to the well-known iNOS inhibitor **1400W** in terms of cytotoxicity and cell responses towards inflammation. Moreover, the capability of these molecules in recovering the mineralization process and enhancing angiogenesis under inflammatory conditions was investigated.

## 2. Results and Discussion

Dental pulp cells interact with immunogenic components such as LPS (lipopolysaccharide) from deep infiltrating carious lesions, and pulp inflammation occurs. Under inflammation, the secretion of cytokines triggered by bacterial products may influence the mineralization potential of mesenchymal stem cells inside the dental pulp (DPSCs). In particular, the LPS-stimulated iNOS induction might have detrimental effects in terms of vital functions of pulp cells such as mineralization, neurogenesis, angiogenesis and neurovascular inductive activity [20], and lead to oxidative/nitrosative stress-induced cell apoptosis [6]. In this light, a selective iNOS inhibition might be beneficial to counteract inflammation in DPSCs. With this aim, a cell model of LPS-stimulated DPSCs was established and the effects of two newly synthetized selective iNOS inhibitors selected from previous investigations [17,18] were evaluated in terms of cytotoxicity, mineralization occurrence, inflammation markers and VEGF secretion. The concentrations used in the present study were chosen based on previously obtained results on human monocytes and pulp cells. In that context, all the concentrations tested were ineffective on DPSCs after 24 and 72 h, whereas doses higher than 50 µM caused cytotoxicity to human monocytes. For this reason and because compounds were here used for up to 21 days, we decided to use concentrations not higher than 50 µM [18].

**Figure 2:**
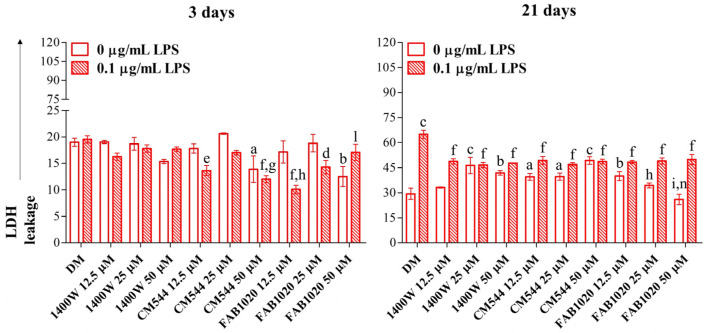
Cytotoxicity in DPSCs after 3 and 21 days. Bar graphs show the LDH released from cells under non-inflammatory (0 µg/mL LPS) and pro-inflammatory conditions (0.1 µg/mL LPS) in an osteogenic environment (DM) in the presence of increasing concentrations of inhibitors (12.5–50 µM). A lysed sample was set as positive lysed control (100%, not shown). DM = differentiation medium. a = *p* < 0.01; b = *p* < 0.001; c = *p* ≤ 0.0001 between samples and DM. d = *p* < 0.01; e = *p* < 0.001; f = *p* ≤ 0.0001 between samples and DM + LPS. g = *p* < 0.01; h = *p* < 0.001; i = *p* ≤ 0.0001 between samples and **1400W** in the same experimental conditions. l = *p* < 0.01; n = *p* ≤ 0.0001 between samples and **CM544** in the same experimental conditions.

To test the biocompatibility of the newly synthetized iNOS inhibitors on DPSCs, inflamed or not, a cytotoxicity assay was performed after early (3 days) and longer exposure times (21 days) and results were compared to those obtained from a **1400W** exposure. After 3 days, DPSCs assessed as control (DM) weakly secrete LDH under osteogenic conditions independently from LPS (around 20% of the lysed positive control). Likewise, when the **1400W** is added to cultures, LDH values are comparable to those of the control sample. In LPS-stimulated DPSCs in the presence of **CM544**, a significant LDH decrease is registered already at 12.5 µM and it is even more amplified at 50 µM. When the **FAB1020** inhibitor is added to cultures, the LDH is slightly secreted both when the LPS is present or not. After 21 days of exposure, the LDH released in LPS-stimulated cells is doubled (64.9%) with respect to the DM alone (29.3%). The **1400W** and **CM544** inhibitors seem to be cytotoxic in a dose-dependent manner when cells are not stimulated with LPS, the LDH leakage assessed at 41.9% and 49.2% for both compounds at the maximum concentration of 50 µM. On the contrary, they can counteract the LPS-induced cytotoxicity, but this effect is independent of the concentration (about 48% for both compounds at all the concentration tested). On the other hand, **FAB1020** significantly decreases cytotoxicity both when the LPS is present or not. More in details, a dose-dependent decrease of secreted LDH is registered up to values comparable with the DM control at 50 µM (26%) without LPS. Contrariwise, when DPSCs are LPS-stimulated, values of LDH are comparable with those in the presence of **1400W** and **CM544** (Figure 2).

**Figure 3:**
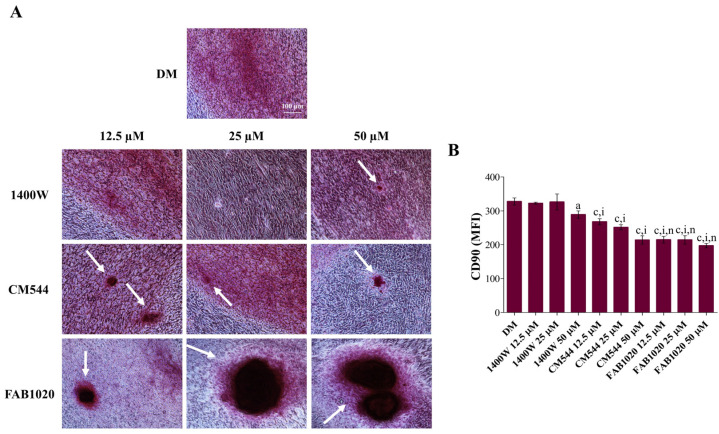
Mineralization occurrence in DPSCs in the presence of iNOS inhibitors. (**A**) Imaging of cell cultures in situ after staining with Alizarin Red. Cells were exposed to increasing concentrations of inhibitors (12.5–50 µM) without LPS for 21 days. DM = differentiation medium. Scale bar = 100 µm. White arrows indicate calcium nodules. (**B**) The bar graph shows the mean fluorescence intensity (MFI) related to the surface marker CD90 in an osteogenic environment (DM) in the presence of increasing concentrations of inhibitors (12.5–50 µM) without LPS after 3 days of exposure. Values are presented as the means ± standard deviations summarized from individual samples in three independent experiments (n = 6). DM = differentiation medium. a = *p* < 0.01; c = *p* ≤ 0.0001 between samples and DM. i = *p* ≤ 0.0001 between samples and **1400W** in the same experimental conditions. n = *p* ≤ 0.0001 between samples and **CM544** in the same experimental conditions.

**Figure 4:**
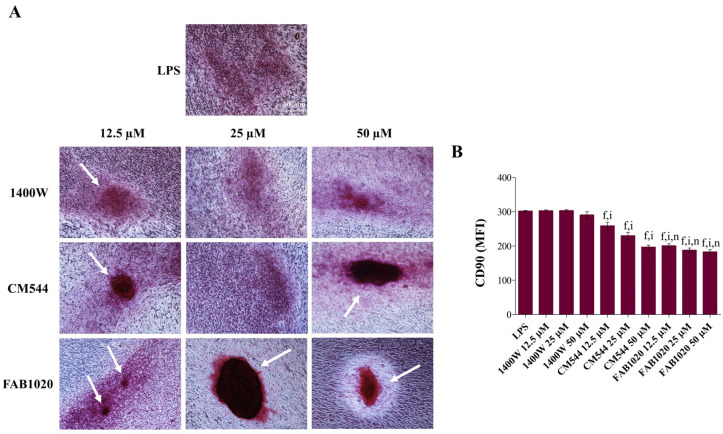
Mineralization occurrence in inflamed DPSCs in the presence of iNOS inhibitors. (**A**) Imaging of cell cultures in situ after staining with Alizarin Red. Cells were exposed to increasing concentrations of inhibitors (12.5–50 µM) in the presence of LPS in the differentiation medium for 21 days. Scale bar = 100 µm. White arrows indicate calcium nodules. (**B**) The bar graph shows the mean fluorescence intensity (MFI) related to the surface marker CD90 in an osteogenic environment (DM) in the presence of increasing concentrations of inhibitors (12.5–50 µM) and LPS after 3 days of exposure. Values are presented as the means ± standard deviations summarized from individual samples in three independent experiments (n = 6). f = *p* ≤ 0.0001 between samples and DM + LPS. i = *p* ≤ 0.0001 between samples and **1400W** in the same experimental conditions. n = *p* ≤ 0.0001 between samples and **CM544** in the same experimental conditions.

Once the biological activity of the selected iNOS inhibitors had been assessed, to investigate the occurrence of the extracellular matrix mineralization, the Alizarin Red staining was performed in all the experimental conditions after 21 days of exposure (Figure 3A and Figure 4A). In parallel, the expression of the stemness-related marker CD90 was assessed by immunophenotyping (Figure 3B and Figure 4B). The dental pulp contains mesenchymal stem cells (MSCs) which express associated markers. Remarkable expression-levels of CD90, CD73, CD105 and CD29 represent the minimal criteria for defining a multipotent mesenchymal stromal cell [21]. It has been already reported that a DPSC exposure to compounds able to act on the antioxidant molecular machinery can modulate CD marker expression and can be crucial in the MSC fate decision in terms of cell stemness loss in favor of modulation of osteogenic gene expression and mineralization occurrence [10]. In particular, the decreased expression of the GPI-anchored cell surface protein CD90 has been linked to the enhancement of the MSC osteogenic differentiation in vitro [22,23]. In our experimental model, under osteogenic conditions and in the absence of LPS (Figure 3A), a strong increase in clusters of calcium and phosphate salts can be observed in all the experimental conditions, mainly in the presence of **CM544** and even more in the presence of **FAB1020** (white arrows). In parallel, expression-levels of CD90 after 3 days of culture (Figure 3B) decrease in a dose-dependent manner in the presence of **1400W** (MFI at 50 µM = 289) with respect to the DM sample (MFI = 328). The addition of **CM544** to cultures already enhances the CD90 fall at 12.5 µM (MFI = 268). In the presence of **FAB1020**, the MFIs related to CD90 are very low, independently of the inhibitor concentration, being all assessed around 200. In the presence of a low concentration of LPS, DPSCs retain their capacity to mineralize, as shown in Figure 4A. As for their non-inflamed counterpart, clusters of calcium and phosphate salts can be observed in all the experimental conditions, mainly in the presence of **CM544** and **FAB1020**. Expression-levels of CD90 (Figure 4B) are also decreased dose-dependently in the presence of **CM544** and independently from the concentrations when the **FAB1020** is present. It is therefore plausible to assume that the observations reported for LPS-stimulated DPSCs in the presence of several antioxidants [10], or rather the stimulation with a low LPS concentration, does not affect the mineralization capacity of pulp cells, are strongly validated. It has already been reported that low concentrations of NO stimulate odontogenic differentiation [24], and LPS is known to be an enhancer in NO production through NfκB activation [25]. In parallel, an exaggerated NFκB activation through massive NO generation produced through the iNOS upregulation has been reported to weaken MSC differentiation [26]. In the presence of the **1400W** inhibitor, despite its selective effect on the iNOS enzyme, to less extent in the extracellular cell matrix (ECM) mineralization can be detected with respect to **CM544** and **FAB1020**, independently from LPS. This could be related to the higher polarity of **1400W** with respect to the other two compounds, as reported in Table 1, which might impair its cell permeation and thus imply a prolonged lag-time before the onset of the biological effect, as previously observed [16].

**Figure 5:**
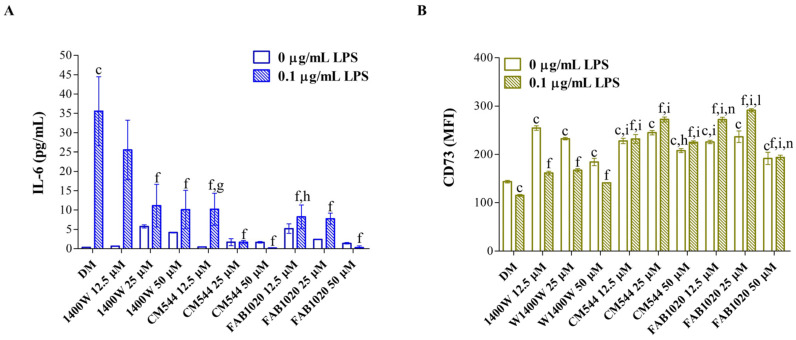
Markers of inflammation in DPSCs after 3 days in the presence of iNOS inhibitors. (**A**) Bars represent mean values ± standard deviations combined from independent experiments (n = 4) related to the secretion of interleukin-6 (IL-6) as pg/mL. (**B**) The bar graph shows the mean fluorescence intensity (MFI) related to the surface marker CD73. Values are presented as the means ± standard deviations summarized from individual samples in three independent experiments (n = 6). c = *p* ≤ 0.0001 between samples and DM. f = *p* ≤ 0.0001 between samples and DM + LPS. g = *p* < 0.01; h = *p* < 0.001; i = *p* ≤ 0.0001 between samples and **1400W** in the same experimental conditions. l = *p* < 0.01; n = *p* ≤ 0.0001 between samples and **CM544** in the same experimental conditions.

In assessing the effects of the three selective iNOS inhibitors on the DPSC mineralization capacity after long exposure times (21 days), markers of inflammation were measured earlier (3 days), to investigate whether the biological effects assessed in terms of mineralization might be related to the anti-inflammatory response triggered by the selected compounds. Firstly, the IL-6 secretion was analyzed (Figure 5A), as this interleukin is gaining increasing interest owing to its dual role, both in inflammation and in odontogenic differentiation, highlighting the possibility of an MSC-mediated immunoregulation [27]. Moreover, expression-levels of the ecto-5′nucleotidase (CD73) were measured (Figure 5B). Beyond its widely reported role in MSC osteoblastic/odontoblastic differentiation [28], the enzymatic activity of CD73 plays a strategic role in calibrating the duration, magnitude and chemical nature of purinergic signals delivered to immune cells through the conversion of ADP/ATP to AMP and AMP to adenosine, respectively [29]. It was reported that the CD73-triggered signal stimulates a shift from an ATP-driven pro-inflammatory environment to an anti-inflammatory niche induced by adenosine [30]. As expected, a LPS exposure dramatically increases the secretion of IL-6 (35.6 pg/mL) from DPSCs with respect to the DM alone (0.3 pg/mL) in our experimental model (Figure 5A). When increasing concentrations of **1400W** were administered, a strong dose-dependent decrease of IL-6 secretion is registered (10.1 pg/mL at 50 µM). Notably, in the presence of **CM544** and **FAB1020**, IL-6 secretion is already slightly detected at 12.5 µM (10.1 pg/mL and 8.2 pg/mL, respectively). In parallel, CD73 is upregulated in all the experimental conditions with respect to DM (MFI = 143.6) and LPS alone (MFI = 115.2). In more detail, in the presence of **1400W** and LPS, MFIs related to CD73 are significantly higher than those of the control sample (MFI at 25 µM = 167.1) but to a lesser extent than in the presence of **CM544** and **FAB1020** (MFIs at 25 µM = 272.4 and 291.2, respectively). It is therefore plausible to speculate that the decrease in IL-6 secretion under LPS-induced pro-inflammatory conditions might be triggered by the CD73, its expression-levels being increased when the IL-6 is weakly secreted. Moreover, this intriguing observation seems to confirm the proposed role in immunomodulation for DPSCs and in general for MSCs.

**Figure 6:**
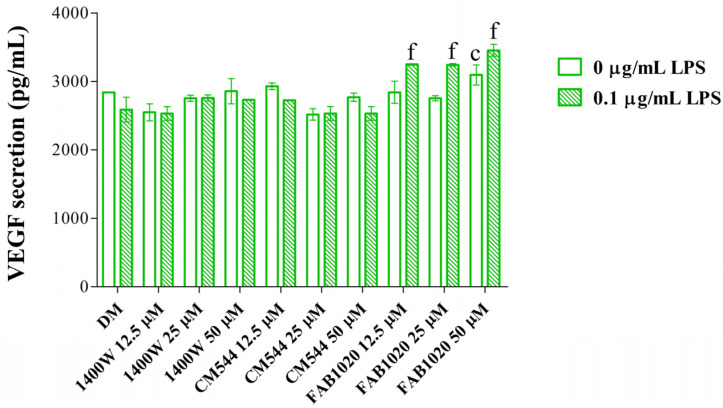
VEGF secreted from DPSCs after 21 days in the presence of iNOS inhibitors. Bars show mean values ± standard deviations combined from independent experiments (n = 4) related to the secretion of vascular endothelial growth factor (VEGF), as pg/mL. c = *p* ≤ 0.0001 between samples and DM. f = *p* ≤ 0.0001 between samples and DM + LPS.

DPSCs exist in the vessel microenvironment in vivo and have a close association with endothelial cells. It has been reported that these cells have a mutual effect on each other, because MSC-induced promotion of tooth regeneration is highly associated with increased angiogenesis, which promotes cell migration and growth factor delivery to the injured site [31,32]. Against this background the secretion of VEGF was measured in all the experimental conditions (Figure 6). In vivo transplantation studies have shown that silencing VEGFR-2 hampers odontoblastic differentiation of DPSCs [33]. Hardly detectable, and thus insignificant, fluctuations can be observed in the presence of **1400W** and **CM544** with respect to the control samples, independently from LPS. On the contrary, **FAB1020** slightly but significantly increases VEGF secretion, mainly in the presence of LPS. These findings are in line with the well-known role of NO in both physiological and pathological angiogenesis [34] and with the fact that a correct modulation of NO production, due to a regulated balance among the three-isoform activity, is necessary for physiological osteogenesis [35].

## 3. Materials and Methods

### 3.1. Synthesis of iNOS Inhibitors

The two selective iNOS inhibitors **CM544** and **FAB1020** were synthesized, purified, and characterized as previously reported [15,16]. **1400W** was purchased from Merck (Darmstadt, Germany). Compounds were stored at −20 °C.

### 3.2. Cell Culture

Dental Pulp Stem Cells (DPSCs) were purchased from Lonza (Lonza Group Ltd., Basel, Switzerland) and sub-cultured in the presence of α-MEM medium (Merck, Darmstadt, Germany) supplemented by 10% FBS and 1% penicillin/streptomycin (Euroclone S.p.A., Milan, Italy) at 37 °C and 5% CO_2_ up to passage 5.

### 3.3. Establishment of Pro-Inflammatory Conditions and Cell Exposures to Inhibitors

DPSCs from routine culture were seeded as passage 6 in complete α-MEM and incubated for 48 h at 37 °C and 5% CO_2_. After that, cells were stimulated by Lipopolysaccharide (LPS from *E. coli*, Merck, Darmstadt, Germany) 0.1 µg/mL to establish an inflamed environment. In parallel, DPSCs were exposed to the iNOS inhibitors **1400W** or **CM544** (stock solutions 50 mM in deionized water) or **FAB1020** (stock solution 50 mM in DMSO) at 12.5, 25 and 50 µM, with or without LPS. All the exposures were performed under odontogenic conditions by diluting the LPS and compounds in complete α-MEM supplemented with 10 nM dexamethasone, 10 mM β-glycerophosphate and 50 µg/mL ascorbic acid (all from Merck, Darmstadt, Germany) as described elsewhere [10]. Fresh exposure media were provided every 72 h.

### 3.4. Lactate Dehydrogenase (LDH) Release

Cells from routine culture were seeded (7 × 10^3^/well; 96-well plates) in 200 µL of routine culture medium (complete α-MEM) and incubated for 48 h at 37 °C and 5% CO_2_. Next, cells were treated as described previously in this section for up to 21 days. To quantify cytotoxicity after 3 and 21 days, supernatants were harvested, kept on ice, and the CytoTox 96^®^ Non-Radioactive Assay (Promega Corporation, Fitchburg, WI, USA) was performed as already reported [36]. The assay quantitatively measures LDH, a stable cytosolic enzyme that is released upon cell lysis. The maximum LDH release control (positive control) was obtained by adding a lysis solution in a well containing untreated DPSCs and processing the obtained supernatant as for the other experimental conditions. The optical density (O.D.) was measured at 490 and 690 nm by means of a multiscan GO microplate spectrophotometer (Thermo Fisher Scientific, Waltham, MA, USA) and the obtained O.D. were normalized with that related to the positive control (lysed cells). Assessment of cytotoxicity was calculated according to the formula: %LDH released = [(A − B)/(C − B)] × 100, with A = LDH activity of sample, B = LDH activity of untreated cells and C = LDH activity of the positive lysed control.

### 3.5. Interleukin-6 Release

Absolute amounts of IL-6 (pg/mL) were quantified in cell culture supernatants from the same wells used for the LDH assay after 3 days of exposure using a commercial ELISA kit (Enzo Life Sciences Inc, Lausen, Switzerland) as reported previously [18]. Cytokine concentrations were calculated from standard curves using the Prism5 software 5.0 (GraphPad, San Diego, CA, USA). Individual values obtained from independent experiments (n = 4) were summarized as means and standard deviations.

### 3.6. Immunophenotype In Vitro by Flow Cytometry

The expression of surface markers in DPSCs was analyzed by flow cytometry. Cells from routine cultures were seeded (5 × 10^4^/well) in 3 mL of complete α-MEM medium in 6-well plates and then they were let to adhere for 48 h. After exposures for 3 days, cells were harvested and incubated with fluorochrome-conjugated antibodies (1:50 dilutions), namely anti-human cluster of differentiation (CD)90-FITC (BD Biosciences, San Diego, CA, USA) and CD73-PE (R & D Systems, Minneapolis, MN, USA) monoclonal antibodies as reported previously [23]. Relative fluorescence emissions of gated cells by forward and side scatter properties (FSC/SSC) were analyzed with the CytExpert software 2.3 (Beckman Coulter, CA, USA) and expressed as mean fluorescence intensities (MFIs). Individual values obtained from independent experiments (n = 6) were summarized as means and standard deviations.

### 3.7. Extracellular Matrix Mineralization (Alizarin Red Staining)

Cells from routine culture were seeded (5 × 10^4^/well) in 3 mL of complete α-MEM medium in 6-well plates and then let to adhere for 48 h. Then, the cultures were treated as described above. The exposure of cultures was stopped after 21 days by discarding the exposure media. Then, the calcium precipitation was detected after Alizarin red staining of cell cultures following an established procedure [37].

### 3.8. ELISA Analysis of VEGF Secretion

After 21 days of treatment, cell supernatants were collected from the 6-well plate used for the alizarin red staining. The VEGF ELISA kit (Enzo Life Sciences, Farmingdale, NY, USA) was used to perform a quantitative determination (pg/mL) of human VEGF released in the medium following the manufacturer’s instructions. VEGF values were obtained by using a standard curve generated with specific standards, provided by the manufacturer. Individual values obtained from independent experiments (n = 4) were summarized as means and standard deviations.

### 3.9. Statistics

Statistical analysis was performed using the GraphPad 5.0 software (GraphPad Software, San Diego, CA, USA), by means of a t-test and ordinary one-way ANOVA, followed by post hoc Tukey’s multiple comparisons tests. Values of *p* < 0.05 were considered statistically significant.

## 4. Conclusions

The present work investigates the effects of two newly synthetized selective iNOS inhibitors, namely **CM544** and **FAB1020**, in a cell model of human DPSCs stimulated by LPS to induce an inflamed environment. Our results were compared to those obtained in the presence of **1400W**, a known selective iNOS inhibitor. The investigation context is intriguing, considering the close link between inflammation and the induction of regenerative processes in the dental pulp. In parallel, this investigation is innovative, due to the lack of pre-clinical data on the potential therapeutic role that selective iNOS inhibitors might play in in vitro models of pulpitis. Our results reveal that **CM544**, and even more **FAB1020**, are effective in decreasing the inflammatory response in DPSCs, by impairing IL-6 secretion and upregulating the MSC marker CD73, an ecto-5′nucleotidase which also has several key functions in cell responses towards inflammation, associated with the shift from an ATP-driven pro-inflammatory environment to an anti-inflammatory niche induced by adenosine. In parallel, the ECM mineralization is retained and further increased in the presence of the two inhibitors, with respect to **1400 W**. Data reported confirm the effectiveness of **CM544** and **FAB1020** and their ameliorated biological profile with respect to **1400 W**. Moreover, by exposing cells to these compounds, knowledge of the biology of DPSCs has been improved, confirming their active role not only in odontogenesis but also in immunomodulation and cell responses towards inflammation. This study lays the ground for future investigations about the role of NO in the dental pulp complex and in cells at the dentin–pulp interface. Moreover, the function of MSCs in the dental pulp as cells modulating immunity under pathogenic stimuli or stressor exposition is worthy of investigation in detail. Based on their effectiveness in the present experimental model, the iNOS selective inhibitors here tested could be studied in cell models of regeneration or in a tissue engineering context.

## Figures and Tables

**Table 1:** IC_50_ values and physico-chemical parameters of selected iNOS inhibitors.

Cpd	iNOS IC_50_ (nM)	eNOS/iNOS Selectivity	logP *	TPSA *
**1400W**	81	3800	1.06	61.90
**CM544**	56	4569	1.31	68.22
**FAB1020**	11	>900	2.70	64.98

* Calculated by the SwissADME online software (http://www.swissadme.ch).

## Data Availability

Data are contained within the article.

## References

[1] Lundberg O.J., Weitzberg E. (2022). Nitric oxide signaling in health and disease. Cell.

[2] Mir J.M., Maurya R.C. (2018). A gentle introduction to gasotransmitters with special reference to nitric oxide: Biological and chemical implications. Rev. Inorg. Chem..

[3] Xue Q., Yan Y., Zhang R., Xiong H. (2018). Regulation of iNOS on Immune Cells and Its Role in Diseases. Int. J. Mol. Sci..

[4] Król M., Kepinska M. (2021). Human Nitric Oxide Synthase—Its Functions, Polymorphisms, and Inhibitors in the Context of In-flammation, Diabetes and Cardiovascular Diseases. Int. J. Mol. Sci..

[5] Galler K., Weber M., Korkmaz Y., Widbiller M., Feuerer M. (2021). Inflammatory Response Mechanisms of the Dentine–Pulp Complex and the Periapical Tissues. Int. J. Mol. Sci..

[6] Schweikl H., Birke M., Gallorini M., Petzel C., Bolay C., Waha C., Hiller K., Buchalla W. (2021). HEMA-induced oxidative stress inhibits NF-κB nuclear translocation and TNF release from LTA- and LPS-stimulated immunocompetent cells. Dent Mater..

[7] Di Nardo Di Maio F., Lohinai Z., D’Arcangelo C., De Fazio P.E., Speranza L., De Lutiis M., Patruno A., Grilli A., Felaco M. (2004). Nitric oxide synthase in healthy and inflamed human dental pulp. J. Dent. Res..

[8] Parwani S.R., Parwani R.N. (2015). Nitric oxide and inflammatory periodontal disease. Gen Dent..

[9] Rothermund K., Calabrese T.C., Syed-Picard F.N. (2022). Differential Effects of *Escherichia coli*—Versus *Porphyromonas gingivalis*—Derived Lipopolysaccharides on Dental Pulp Stem Cell Differentiation in Scaffold-free Engineered Tissues. J. Endod..

[10] Gallorini M., Widbiller M., Bolay C., Carradori S., Buchalla W., Cataldi A., Schweikl H. (2022). Relevance of Cellular Redox Ho-meostasis for Vital Functions of Human Dental Pulp Cells. Antioxidants.

[11] An S. (2020). Nitric Oxide in Dental Pulp Tissue: From Molecular Understanding to Clinical Application in Regenerative Endodontic Procedures. Tissue Eng. Part B Rev..

[12] Sobrinho J.E.M., Aguiar M.T., Machado L.C., Carlos A.C.A.M., Nunes Alves A.P.N., Mesquita K.C., de Miranda Candeiro G.T., de Barros Silva P.G. (2022). Intense orthodontic force induces the three dental pulp nitric oxide synthase isoforms and leads to orofacial discomfort in rats. Orthod. Craniofacial Res..

[13] Donnini S., Ziche M. (2002). Constitutive and Inducible Nitric Oxide Synthase: Role in Angiogenesis. Antioxidants Redox Signal..

[14] Cinelli M.A., Do H.T., Miley G.P., Silverman R.B. (2019). Inducible nitric oxide synthase: Regulation, structure, and inhibition. Med. Res. Rev..

[15] Maccallini C., Di Matteo M., Gallorini M., Montagnani M., Graziani V., Ammazzalorso A., Amoia P., De Filippis B., Di Silvestre S., Fantacuzzi M. (2018). Discovery of N-{3-[(ethanimidoylamino)methyl]benzyl}-l-prolinamide dihydrochloride: A new potent and selective inhibitor of the inducible nitric oxide synthase as a promising agent for the therapy of malignant glioma. Eur. J. Med. Chem..

[16] Maccallini C., Arias F., Gallorini M., Amoia P., Ammazzalorso A., De Filippis B., Fantacuzzi M., Giampietro L., Cataldi A., Camacho M.E. (2020). Antiglioma Activity of Aryl and Amido-Aryl Acetamidine Derivatives Targeting iNOS: Synthesis and Biological Evaluation. ACS Med. Chem. Lett..

[17] Gallorini M., Maccallini C., Ammazzalorso A., Amoia P., De Filippis B., Fantacuzzi M., Giampietro L., Cataldi A., Amoroso R. (2019). The Selective Acetamidine-Based iNOS Inhibitor CM544 Reduces Glioma Cell Proliferation by Enhancing PARP-1 Cleavage In Vitro. Int. J. Mol. Sci..

[18] Gallorini M., Rapino M., Schweikl H., Cataldi A., Amoroso R., Maccallini C. (2021). Selective Inhibitors of the Inducible Nitric Oxide Synthase as Modulators of Cell Responses in LPS-Stimulated Human Monocytes. Molecules.

[19] Grottelli S., Amoroso R., Macchioni L., D’Onofrio F., Fettucciari K., Bellezza I., Maccallini C. (2020). Acetamidine-Based iNOS Inhibitors as Molecular Tools to Counteract Inflammation in BV2 Microglial Cells. Molecules.

[20] Sui B., Chen C., Kou X., Li B., Xuan K., Shi S., Jin Y. (2018). Pulp Stem Cell–Mediated Functional Pulp Regeneration. J. Dent. Res..

[21] Dominici M., Le Blanc K., Mueller I., Slaper-Cortenbach I., Marini F.C., Krause D.S., Deans R.J., Keating A., Prockop D.J., Horwitz E.M. (2006). Minimal criteria for defining multipotent mesenchymal stromal cells. The International Society for Cellular Therapy position statement. Cytotherapy.

[22] Moraes D.A., Sibov T.T., Pavon L.F., Alvim P.Q., Bonadio R.S., Da Silva J.R., Pic-Taylor A., Toledo O.A., Marti L.C., Azevedo R.B. (2016). A reduction in CD90 (THY-1) expression results in increased differentiation of mesenchymal stromal cells. Stem Cell Res. Ther..

[23] Gallorini M., Di Carlo R., Pilato S., Ricci A., Schweikl H., Cataldi A., Fontana A., Zara S. (2021). Liposomes embedded with differentiating factors as a new strategy for enhancing DPSC osteogenic commitment. Eur. Cells Mater..

[24] Sonoda S., Mei Y.-F., Atsuta I., Danjo A., Yamaza H., Hama S., Nishida K., Tang R., Kyumoto-Nakamura Y., Uehara N. (2018). Exogenous nitric oxide stimulates the odontogenic differentiation of rat dental pulp stem cells. Sci. Rep..

[25] Napetschnig J., Wu H. (2013). Molecular basis of NF-κB signaling. Annu. Rev. Biophys..

[26] Qin A., Chen S., Wang P., Huang X., Zhang Y., Liang L., Du L.-R., Lai D.-H., Ding L., Yu X. (2021). Knockout of NOS2 Promotes Adipogenic Differentiation of Rat MSCs by Enhancing Activation of JAK/STAT3 Signaling. Front. Cell Dev. Biol..

[27] Philipp D., Suhr L., Wahlers T., Choi Y.-H., Paunel-Görgülü A. (2018). Preconditioning of bone marrow-derived mesenchymal stem cells highly strengthens their potential to promote IL-6-dependent M2b polarization. Stem Cell Res. Ther..

[28] Takedachi M., Oohara H., Smith B.J., Iyama M., Kobashi M., Maeda K., Long C.L., Humphrey M.B., Stoecker B.J., Toyosawa S. (2011). CD73-generated adenosine promotes osteoblast differentiation. J. Cell. Physiol..

[29] Antonioli L., Pacher P., Vizi E.S., Haskó G. (2013). CD39 and CD73 in immunity and inflammation. Trends Mol. Med..

[30] Allard B., Longhi M.S., Robson S.C., Stagg J. (2017). The ectonucleotidases CD39 and CD73: Novel checkpoint inhibitor targets. Immunol. Rev..

[31] Takeuchi R., Katagiri W., Endo S., Kobayashi T. (2019). Exosomes from conditioned media of bone marrow-derived mesenchymal stem cells promote bone regeneration by enhancing angiogenesis. PLoS ONE.

[32] Rapino M., Di Valerio V., Zara S., Gallorini M., Marconi G.D., Sancilio S., Marsich E., Ghinassi B., di Giacomo V., Cataldi A. (2019). Chit-lac-coated Thermosets Enhance Osteogenesis and Angiogenesis in a Co-culture of Dental Pulp Stem Cells and Endothelial Cells. Nanomaterials.

[33] Janebodin K., Chavanachat R., Hays A., Gil M.R. (2021). Silencing VEGFR-2 Hampers Odontoblastic Differentiation of Dental Pulp Stem Cells. Front. Cell Dev. Biol..

[34] Ziche M., Morbidelli L. (2000). Nitric Oxide and Angiogenesis. J. Neuro-Oncology.

[35] Saura M., Tarin C., Zaragoza C. (2010). Recent insights into the implication of nitric oxide in osteoblast differentiation and prolif-eration during bone development. Sci. World J..

[36] Gallorini M., Zara S., Ricci A., Mangano F.G., Cataldi A., Mangano C. (2021). The Open Cell Form of 3D-Printed Titanium Im-proves Osteconductive Properties and Adhesion Behavior of Dental Pulp Stem Cells. Materials.

[37] Gallorini M., Krifka S., Widbiller M., Schröder A., Brochhausen C., Cataldi A., Hiller K.-A., Buchalla W., Schweikl H. (2020). Distinguished properties of cells isolated from the dentin-pulp interface. Ann. Anat..

